# The Miller General Hospital

**Published:** 1916-05-13

**Authors:** Leonard Miller

**Affiliations:** Acting Chairman.


*To the Editor of* The Hospital.

### The Miller General Hospital.

*To the Editor of* The Hospital.

Sir,—Through the generosity of Mrs. Almeric Paget, this hospital (the Miller General Hospital for South-East London) has been enabled very largely to extend its massage and electrical department and so further to increase its beneficent work.

It may already be known that the "Almeric Paget Massage Corps" (founded by Mr. and Mrs. Paget) is doing all the massage work in the military hospitals and camps. The organisation of the work at these and other places under military control has been carried out by the Hon. Essex French, the daughter of Field-Marshal Viscount French, and it is a source of much gratification to us that Miss French is supervising the starting of the department at this hospital. Previously we had only been able to deal with quite a small number, comparatively, of those patients who required massage and electrical treatment, but now through Mrs. Paget's great kindness the hospital finds itself in a position to attend at once to an almost unlimited number of civilians and wounded and discharged soldiers.

Mrs. Paget is not only providing six masseuses to attend at the hospital daily (except Sundays), but in addition two or more to attend those patients in their own homes who in the opinion of our doctor are not fit to come to the hospital. The department is also being equipped at Mrs. Paget's expense with the necessary appliances for the many forms of treatment that are to be given, including massage, electric vibrations, radiant heat baths, ionisation, galvanism, faradism, breathing exercises, general strengthening and gymnastic treatment, high frequency, electric baths, etc.

No patients who are unable to afford the usual fees for such treatment will be refused, but it is Mrs. Paget's expressed wish that they shall only be treated in the department after they have been approved by the medical officer in charge as being likely to benefit by it.—I am, dear Sir, yours faithfully, Leonard Miller,

May 3, 1916. Acting Chairman.

